# Application of a MRI model based on tumor microenvironment habitat and peritumoral features in preoperative differentiation of rectal cancer T1-2/T3: a multicenter study

**DOI:** 10.3389/fmed.2026.1775773

**Published:** 2026-03-26

**Authors:** Liang Zhang, Dacheng Li, Xiaodan Zhao, Guohua Wang, Xueting Qu

**Affiliations:** 1 Nuclear Medicine Department, The Affiliated Hospital of Qingdao University, Qingdao, Shandong, China; 2 Radiology Department, Qingdao Municipal Hospital, Qingdao University, Qingdao, Shandong, China; 3 Radiology Department, The Affiliated Hospital of Qingdao University, Qingdao, Shandong, China; 4 Radiology Department, Qingdao Traditional Chinese Medicine Hospital, Qingdao Hiser Hospital Affiliated of Qingdao University, Qingdao, Shandong, China

**Keywords:** habitat, MRI, radiomics, rectal cancer, T staging

## Abstract

**Aim:**

To develop a MRI model integrating tumor microenvironment (TME)-driven habitat analysis, peritumoral radiomics, and clinical risk factors for improving preoperative differentiation of T1-2 and T3 stages in rectal cancer.

**Methods:**

This retrospective multicenter study included 313 patients (training cohort: 183; external test cohort: 130). MRI-derived intratumoral radiomic features were clustered into three TME habitats using K-means, while peritumoral features (1 mm, 2 mm, 3 mm extensions) were extracted. Feature selection and model construction were performed using LASSO regression and cross-validation, with all steps strictly confined to the training cohort. Four models were evaluated: peritumoral (PERI2mm optimal), habitat, clinical, and a nomogram combining radiomic features (habitat and PERI2mm) with clinical predictors. Performance was assessed using ROC curves, calibration metrics (intercept, slope, Brier score), and decision curve analysis (DCA).

**Results:**

The nomogram achieved the highest AUC of 0.907 (training) and 0.881 (test), outperforming standalone models in the test cohort (PERI2mm: AUC = 0.721; habitat: AUC = 0.741; clinical: AUC = 0.723). The nomogram also demonstrated superior balanced accuracy (0.827), PR-AUC (0.948), and F1 score (0.851) in the test cohort. Key clinical predictors included age, elevated CEA, tumor length, circumferential growth and mrT stage. Calibration analysis confirmed excellent agreement between predicted and pathological staging, with the nomogram showing the lowest Brier scores (0.1214 training, 0.1253 test) and intercepts near zero, indicating no significant systematic bias. DCA demonstrated superior clinical net benefit across risk thresholds.

**Conclusion:**

The TME-integrated nomogram significantly enhances preoperative T1-2/T3 staging accuracy in rectal cancer by leveraging habitat heterogeneity, peritumoral radiomics, and clinical biomarkers, with robust calibration and generalization. This tool may refine therapeutic strategies, reduce overtreatment, and improve patient outcomes.

## Introduction

Colorectal cancer ranks as the third most common malignancy globally but holds the second position in mortality (1). Accurate preoperative T staging of rectal cancer, particularly distinguishing T1-2 from T3 stages, is pivotal for treatment decision-making. According to the NCCN guidelines, T3 patients require neoadjuvant chemoradiotherapy followed by surgery, whereas T1-2 patients may proceed directly to resection to avoid overtreatment (2). High-resolution MRI remains the primary modality for preoperative staging (3, 4); however, its limitations in detecting subtle extramural invasion or desmoplastic reactions in T3 tumors result in the overall diagnostic accuracy of MRI is 66–88% (5–7). The overall performance of MRI in staging early rectal cancer was disappointing (8). These diagnostic uncertainties may lead to clinical staging errors, potentially causing overtreatment or undertreatment (9).

Recent advances in radiomics, which involve high-throughput extraction of tumor heterogeneity features, offer a paradigm shift beyond conventional visual imaging assessments (10, 11). However, existing studies predominantly focus on intratumoral texture analysis, lacking granular exploration of tumor subregions (e.g., necrotic cores, invasive fronts) and the tumor microenvironment (TME). Spatial heterogeneity within the TME—such as stromal remodeling, immune cell infiltration, and angiogenesis—has been strongly linked to tumor aggressiveness and staging (12). Emerging evidence suggests that TME-driven biomarkers could refine imaging-based prognostication (12).

Habitat analysis provides an innovative solution by segmenting tumors into biologically distinct subregions (e.g., hypervascular or hypoxic zones) and quantifying spatial heterogeneity patterns (13–15). Studies have demonstrated that, compared to traditional radiomics, habitat analysis can more accurately and comprehensively quantify tumor heterogeneity, thereby establishing more reliable imaging biomarkers for personalized treatment (16). Although habitat-based radiomics as shown potential in tumor diagnosis, staging, and treatment response assessment (17, 18), its value in distinguishing between T1-2 and T3 stages of rectal cancer remains to be elucidated.

This study aims to develop a TME-driven MRI model that integrates habitat analysis, peritumoral radiomics, and clinical risk factors to improve staging accuracy. The findings are expected to provide novel biomarkers for personalized therapeutic strategies in rectal cancer.

## Materials and methods

### Ethical approval and study design

**Figure 1:**
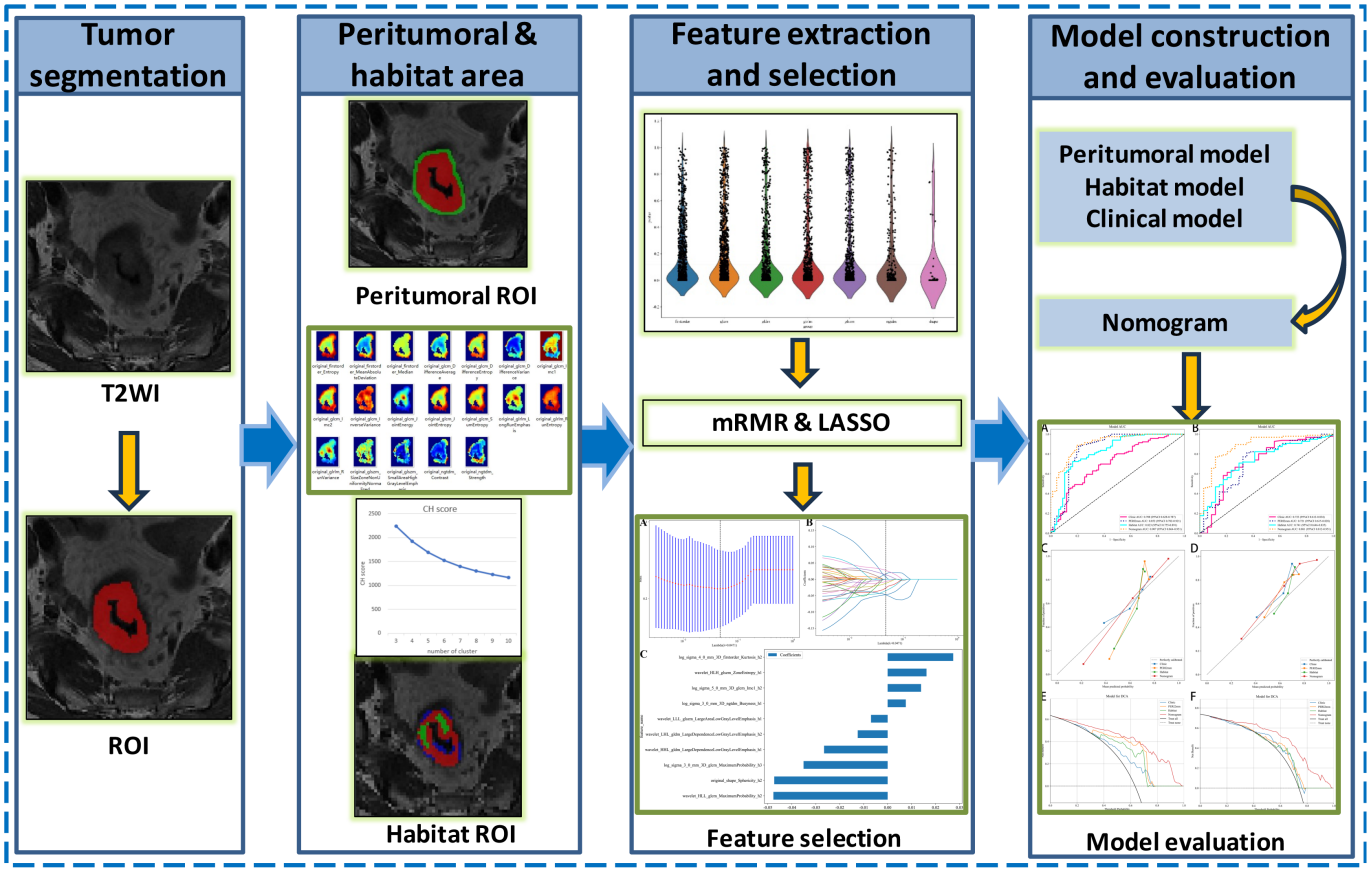
The flowchart of this study.

This study was approved by the Ethics Committee of the Affiliated Hospital of Qingdao University (QYFY WZLL 30246). Written informed consent was obtained from all patients prior to their inclusion in the study, authorizing the use of their clinical and imaging data for research purposes. Data were collected from four medical centers (Supplementary S1): patients from Center A were assigned to the training cohort (*n* = 183), while those from other 3 Centers formed the external test cohort (*n* = 130). Inclusion criteria: (1) pathologically confirmed rectal adenocarcinoma treated with radical resection; (2) preoperative high-resolution MRI performed within 2 weeks before surgery. Exclusion criteria: (1) pathological T4 stage; (2) incomplete clinical or imaging data; (3) poor-quality MRI images; (4) receipt of any neoadjuvant therapy (including chemoradiotherapy, radiotherapy, or chemotherapy) prior to surgery. The study flowchart is illustrated in Figure 1.

### Clinical and imaging characteristics

Collected variables included age, sex, carcinoembryonic antigen (CEA), CA19-9, distance from the tumor to the anus (Dis), depth of invasion, tumor length, mrTN stage, distant metastasis (M), circumferential growth (Cir), mesorectal fascia (MRF), and extramural vascular invasion (EMVI). Patients were stratified into T1-2 and T3 groups based on postoperative pathological staging.

### MRI acquisition and preprocessing

MRI protocols include axial T1-weighted imaging (T1WI), axial/sagittal T2-weighted imaging (T2WI), and diffusion-weighted imaging (DWI). Patients were scanned in the supine position without bowel preparation. Axial T2WI images (The parameters were detailed in Supplementary S1) were exported from the Picture Archiving and Communication System (PACS) and preprocessed using 3D Slicer (v5.0.3): N4 bias field correction to address magnetic field inhomogeneity, grayscale normalization to minimize signal intensity variations, and all images were resampled to 1 × 1 × 1 mm^3^ isotropic voxels using nearest-neighbor interpolation.

### Tumor segmentation and feature extraction

Two radiologists (with 6 and 15 years of experience in rectal cancer imaging) independently and blindly delineated the tumor region of interest (ROI) on axial T2WI slice-by-slice to generate volumetric ROIs (VOIs). Both radiologists were blinded to the pathological staging and clinical outcomes during the segmentation process. Discrepancies between the two independent segmentations were resolved through consensus, and the consensus segmentations were used for subsequent feature extraction and model construction. Thirty randomly selected cases were re-annotated after 1 month to calculate intra- and interobserver intraclass correlation coefficients (ICCs); features with ICC ≥ 0.75 were retained (Supplementary S2). Tumor VOIs were automatically expanded by 1 mm, 2 mm, and 3 mm using OnekeyAI software, with manual removal of luminal components, yielding peritumoral regions (PERI1mm, PERI2mm, PERI3mm). PyRadiomics was used to extract shape, intensity, and texture features from these regions. The PyRadiomics parameter file was shown in Supplementary S3.

### Habitat generation

**Figure 2:**
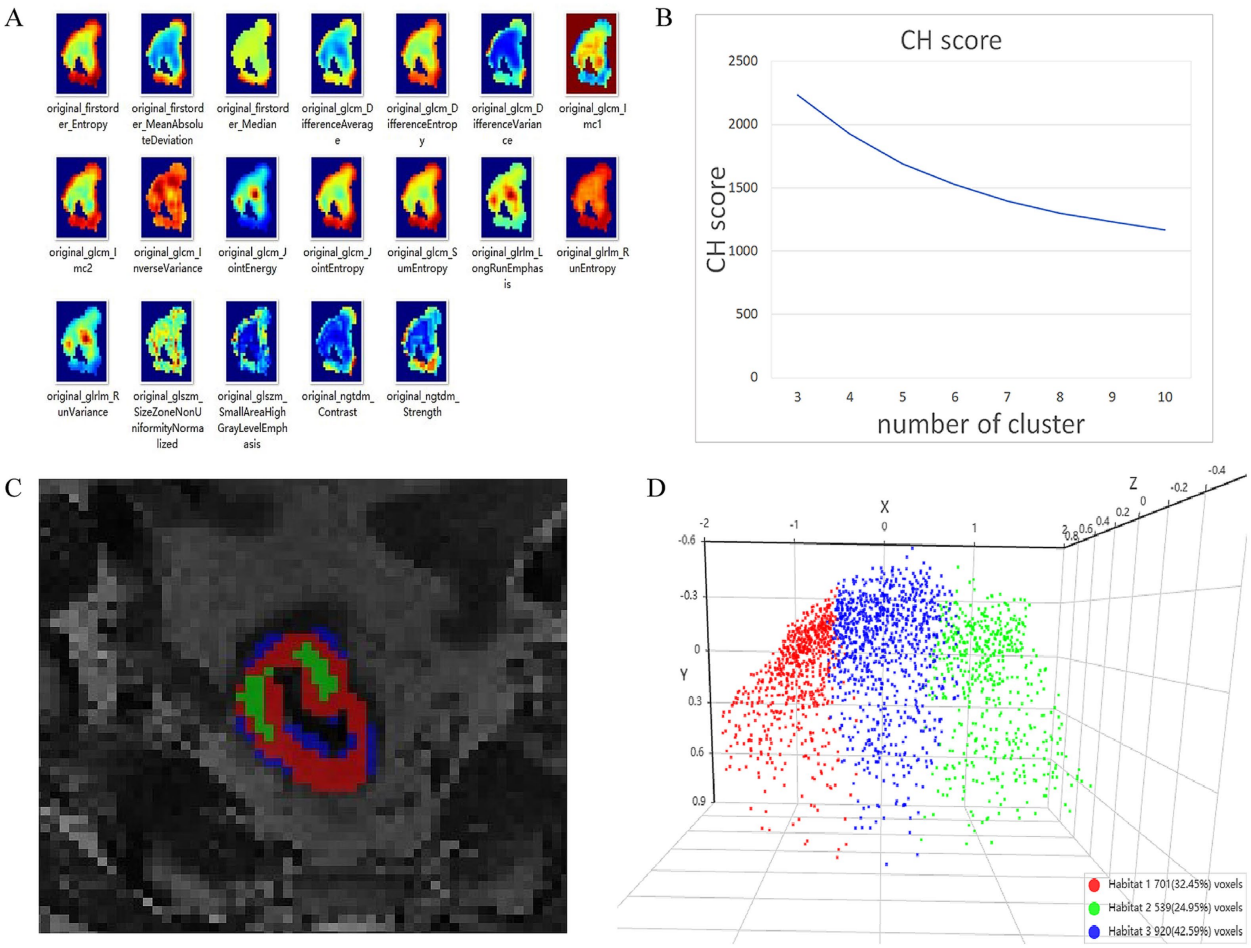
The visualized radiomic feature heatmap of each voxel in the tumor **(A)**. The CH score curve shows that the CH score is the highest when the number of clusters was 3 **(B)**. Therefore, the tumor area is divided into three habitat regions **(C)**. The 3D visualization figure of the radiomics features extracted from three habitat regions, respectively **(D)**.

Tumor VOIs were resampled to 2mm^3^ voxels, and voxel-wise radiomic features were extracted using PyRadiomics (Supplementary S4). For each patient, K-means clustering with the number of clusters ranging from 3 to 10 was applied to partition the tumor into biologically distinct habitats. The optimal cluster number (n = 3) was determined using the Calinski-Harabasz (CH) index (Supplementary S5), resulting in three habitat regions (feature_h1, feature_h2, feature_h3) as shown in Figure 2.

### Feature selection and model construction evaluation

All feature selection steps were strictly confined to the training cohort. Features were normalized via *z*-score transformation. Robust features were selected using ICC > 0.75 and Spearman correlation. The minimum redundancy maximum relevance (mRMR) algorithm retained 40 low-redundancy features. Least Absolute Shrinkage and Selection Operator (LASSO) regression with 10-fold cross-validation identified non-zero coefficient features, weighted to generate radiomics scores. For each feature set, we performed 10-fold cross-validation on the training set to evaluate the performance of three classifiers (LR, SVM, and MLP), and selected the optimal classifier based on the validation AUC. The hyperparameter details of classifiers were provided in the Supplementary S6. Four models were constructed: (1) peritumoral model (PERI1mm/2 mm/3 mm); (2) habitat model (fused subregional features); (3) clinical model (high-risk factors selected via logistic regression); (4) combined nomogram integrating optimal peritumoral, habitat, and clinical features. Diagnostic performance was assessed using receiver operating characteristic (ROC) curves (area under the curve, AUC), accuracy, sensitivity, specificity, positive predictive value (PPV), and negative predictive value (NPV). Calibration curves and decision curve analysis (DCA) evaluated prediction consistency and clinical net benefit.

### Statistical analysis

Statistical analyses were performed in Python 3.8: normality assessed via Kolmogorov–Smirnov test; features compared using *t*-test/Wilcoxon test; categorical variables analyzed via chi-square test; univariate/multivariate logistic regression identified clinical risk factors; Hosmer–Lemeshow test evaluated model goodness-of-fit. *p*-values less than 0.05 indicate statistical significance difference.

## Results

### Patient characteristics

A total of 313 patients from four centers were included according to the inclusion criteria, with 183 patients in the training cohort and 130 in the external test cohort. The clinical baseline characteristics of the cohorts are summarized in Table 1. Among them, 212 patients (67.7%) were classified as pathological T3 stage.

**Table 1:** The clinical baseline characteristics of the patients.

Characteristic	Training cohort		*p* value	Test cohort		*p* value
	T1-2 (*N* = 67)	T3 (*N* = 116)		T1-2 (*N* = 34)	T3 (*N* = 96)	
Age (years)	63.06 ± 11.93	64.21 ± 9.60	0.771	62.00 ± 9.53	64.91 ± 9.21	0.12
Dis (mm)	73.43 ± 32.78	80.98 ± 32.07	0.078	65.82 ± 27.80	76.85 ± 32.01	0.077
Length (mm)	38.76 ± 13.84	46.75 ± 12.36	<0.001	36.68 ± 10.92	45.41 ± 12.29	<0.001
Depth (mm)	1.85 ± 1.09	5.66 ± 4.51	<0.001	1.86 ± 0.75	3.84 ± 3.71	<0.001
Sex						
Female	27 (40.30)	43 (37.07)	0.783	15 (44.12)	32 (33.33)	0.359
Male	40 (59.70)	73 (62.93)		19 (55.88)	64 (66.67)	
Cir						
Negative	48 (71.64)	35 (30.17)	<0.001	27 (79.41)	30 (31.25)	<0.001
Positive	19 (28.36)	81 (69.83)		7 (20.59)	66 (68.75)	
mrT						
1	8 (11.94)	1 (0.86)	<0.001	1 (2.94)	1 (1.04)	<0.001
2	26 (38.81)	5 (4.31)		22 (64.71)	6 (6.25)	
3	30 (44.78)	102 (87.93)		11 (32.35)	79 (82.29)	
4	3 (4.48)	8 (6.90)		0	10 (10.42)	
mrN						
0	49 (73.13)	53 (45.69)	<0.001	17 (50.00)	29 (30.21)	0.029
1	15 (22.39)	39 (33.62)		15 (44.12)	44 (45.83)	
2	3 (4.48)	24 (20.69)		2 (5.88)	23 (23.96)	
M						
Negative	67 (100.00)	109 (93.97)	0.099	33 (97.06)	93 (96.88)	1.000
Positive	0	7(6.03)		1 (2.94)	3 (3.12)	
MRF						
Negative	66 (98.51)	93 (80.17)	<0.001	34 (100.00)	74 (77.08)	0.005
Positive	1 (1.49)	23 (19.83)		0	22 (22.92)	
EMVI						
Negative	65 (97.01)	90 (77.59)	<0.001	31 (91.18)	71 (73.96)	0.036
Positive	2 (2.99)	26 (22.41)		3 (8.82)	25 (26.04)	
CEA						
Negative	56 (83.58)	59 (50.86)	<0.001	30 (88.24)	66 (68.75)	0.026
Positive	11 (16.42)	57 (49.14)		4 (11.76)	30 (31.25)	
CA19-9						
Negative	62 (92.54)	104 (89.66)	0.518	32 (94.12)	85 (88.54)	0.549
Positive	5 (7.46)	12 (10.34)		2 (5.88)	11 (11.46)	

Dis, distance from the tumor to the anus; Cir, circumferential growth; mrT, T stage based on MR; mrN, N stage based on MR; M, metastasis; MRF, mesorectal fascia; EMVI, extramural vascular invasion; CEA, carcinoembryonic antigen; CA19-9, carbohydrate antigen 19–9.

### Construction and selection of the optimal model around the tumor

A total of 1,197 features were extracted from each of the three peritumoral VOIs (1 mm, 2 mm, and 3 mm). After feature selection using LASSO regression, non-zero coefficient features were retained and incorporated into three machine learning classifiers (LR, SVM, MLP) to construct peritumoral radiomics models. The final selected feature names and their coefficients was shown in the calculation formula for each radiomics score (Supplementary S7). As shown in Table 2, the SVM-based peritumoral 2 mm model achieved the highest diagnostic performance in the test cohort (AUC = 0.721), outperforming the 1 mm (AUC = 0.665) and 3 mm (AUC = 0.700) models. Thus, the peritumoral 2 mm model was selected as the optimal peritumoral predictor.

**Table 2:** The diagnostic efficacy of different radiomics models around tumors constructed based on three algorithms.

Model	Classifier	Accuracy	AUC	95% CI	Sensitivity	Specificity	Cohort
PERI1mm	LR	0.645	0.740	0.6664–0.8130	0.543	0.821	Training
	LR	0.685	0.640	0.5216–0.7584	0.698	0.647	Test
	SVM	0.831	0.842	0.7733–0.9107	0.862	0.776	Training
	SVM	0.615	0.665	0.5566–0.7731	0.594	0.676	Test
	MLP	0.689	0.755	0.6834–0.8267	0.681	0.701	Training
	MLP	0.631	0.626	0.5065–0.7453	0.615	0.676	Test
PERI2mm	LR	0.787	0.808	0.7413–0.8751	0.871	0.642	Training
	LR	0.792	0.699	0.5864–0.8125	0.885	0.529	Test
	SVM	0.847	0.852	0.7823–0.9212	0.879	0.791	Training
	SVM	0.769	0.721	0.6148–0.8276	0.812	0.647	Test
	MLP	0.760	0.816	0.7504–0.8816	0.759	0.761	Training
	MLP	0.769	0.683	0.5655–0.8003	0.854	0.529	Test
PERI3mm	LR	0.754	0.839	0.7797–0.8974	0.741	0.776	Training
	LR	0.715	0.707	0.5960–0.8176	0.729	0.676	Test
	SVM	0.852	0.886	0.8280–0.9443	0.888	0.791	Training
	SVM	0.700	0.703	0.5980–0.8071	0.719	0.647	Test
	MLP	0.770	0.837	0.7781–0.8953	0.819	0.687	Training
	MLP	0.623	0.696	0.5941–0.7987	0.573	0.765	Test

### Construction of the habitat model

**Figure 3:**
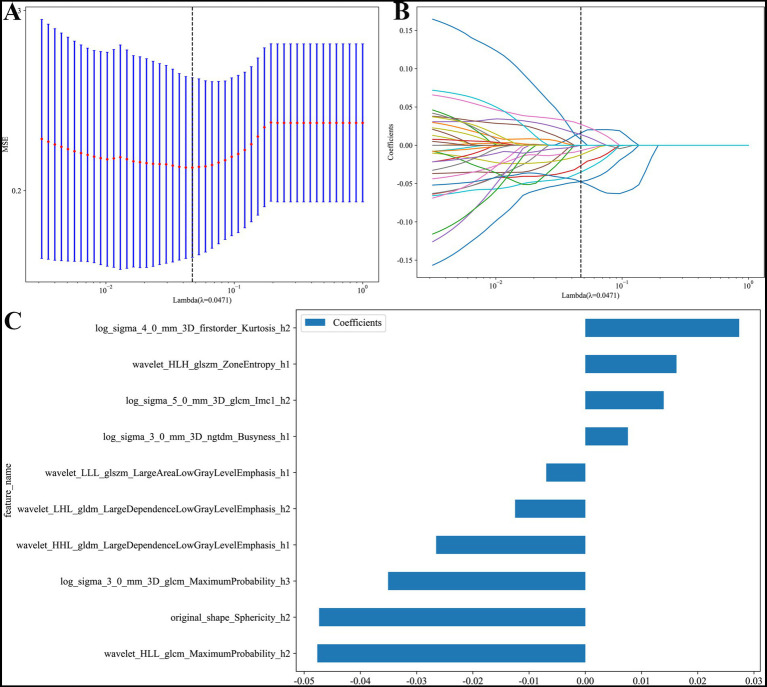
After LASSO regression **(A,B)** screening, 10 habitat features **(C)** were selected from the radiomics characteristics of these habitat areas.

A total of 3,591 features were extracted from the three tumor habitats (feature_h1, feature_h2, feature_h3). LASSO regression identified 10 non-zero coefficient features (Figure 3), which were weighted and incorporated into classifiers. Among the three algorithms, the SVM-based habitat model demonstrated superior performance (Table 3).

**Table 3:** The diagnostic efficacy of the habitat model constructed based on three algorithms.

Classifier	Accuracy	AUC	95% CI	Sensitivity	Specificity	Cohort
LR	0.749	0.793	0.7268–0.8589	0.776	0.701	Training
LR	0.731	0.736	0.6385–0.8339	0.771	0.618	Test
SVM	0.765	0.823	0.7551–0.8913	0.724	0.836	Training
SVM	0.708	0.741	0.6463–0.8353	0.708	0.706	Test
MLP	0.760	0.779	0.7104–0.8470	0.862	0.582	Training
MLP	0.731	0.725	0.6228–0.8276	0.760	0.647	Test

### Construction of the clinical model

Univariate and multivariate logistic regression identified five clinical predictors significantly associated with T3 stage: age (OR = 0.97), CEA (OR = 4.27), length (OR = 0.96), mrT stage (OR = 3.03), and Cir (OR = 3.11). These predictors were integrated into the clinical model (Table 4).

**Table 4:** Multivariate logistic regression analysis screened the clinical model predictive factors.

Characteristic	*β*	OR	95%CI for OR	*p* value
Age	−0.031	0.970	0.948–0.991	0.020
CEA	1.452	4.270	1.895–9.631	0.003
Length	−0.038	0.963	0.934–0.993	0.044
mrT	1.109	3.031	1.749–5.254	0.001
Cir	1.134	3.109	1.439–6.713	0.015
Dis	−0.003	0.997	0.987–1.007	0.609
Depth	0.122	1.130	0.963–1.326	0.211
sex	−0.158	0.854	0.452–1.611	0.682
mrN	0.157	1.170	0.632–2.168	0.675
EMVI	0.884	2.422	0.537–10.924	0.334
MRF	0.769	2.158	0.306–15.196	0.517

### Construction of the combined model (nomogram)

**Figure 4:**
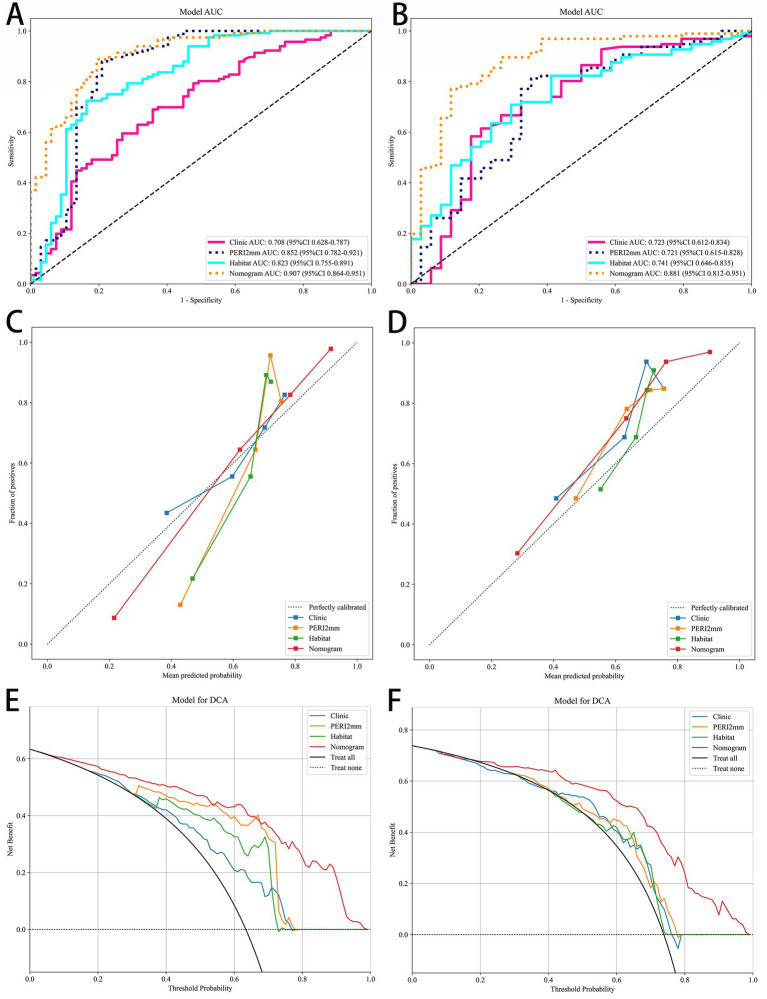
The ROC curves of the four models in the training **(A)** and test **(B)** cohorts; the AUC of the combined model is the highest. Calibration curves of the combined model in the training **(C)** and test **(D)** cohorts; demonstrating good agreement between the predictions and observations. Decision curve analysis (DCA) for predicting T3 stage in the training **(E)** and test **(F)** cohorts; the combined model indicates better clinical net profit.

The nomogram, integrating habitat features, peritumoral 2 mm radiomics features, and clinical predictors, achieved the highest diagnostic performance (Table 5, Figure 4). The Nomogram model performed best in the test set, achieving an AUC of 0.881, balanced accuracy of 0.827, PR-AUC of 0.948, and F1 score of 0.851. The PERI2mm and Habitat model demonstrated moderate performance. The Clinic model exhibited the weakest overall performance. The results of Delong test were shown in Supplementary S8. The calibration curves demonstrated excellent agreement between predicted and pathological staging (Table 6, Figure 4). The Nomogram model exhibited the best calibration performance in both the training and test sets, with its 95% confidence intervals containing zero, indicating no significant systematic bias. The slope was slightly elevated but acceptable, and the Brier scores were the lowest (0.1214 and 0.1253), demonstrating optimal agreement between predicted probabilities and actual outcomes, outperforming the other single models. The decision curve analysis (DCA) confirmed its superior clinical net benefit across risk thresholds (Figure 4).

**Table 5:** Diagnostic efficacy of four models.

Model	Accuracy	AUC	Sensitivity	Specificity	Balanced accuracy	PR-AUC	F1	Cohort
Clinic	0.672	0.708	0.690	0.642	0.666	0.782	0.727	Training
PERI2mm	0.847	0.852	0.879	0.791	0.835	0.853	0.879	Training
Habitat	0.765	0.823	0.724	0.836	0.780	0.842	0.796	Training
Nomogram	0.858	0.907	0.888	0.806	0.847	0.942	0.888	Training
Clinic	0.662	0.723	0.615	0.794	0.705	0.827	0.728	Test
PERI2mm	0.769	0.721	0.812	0.647	0.730	0.853	0.839	Test
Habitat	0.708	0.741	0.708	0.706	0.707	0.890	0.782	Test
Nomogram	0.800	0.881	0.771	0.882	0.827	0.948	0.851	Test

AUC, area under the curve; PR-AUC, precision-recall area under the curve.

**Table 6:** Calibration metrics of the models in training and test cohorts.

Model	Intercept	95% CI (Intercept)	Slope	Brier Score	Cohort
Nomogram	−0.4089	−1.0481 to 0.0754	1.7008	0.1214	Training
Nomogram	0.4957	−0.1222 to 1.0014	1.4005	0.1253	Test
Clinic	0.0222	−0.4002 to 0.4132	1.1824	0.2013	Training
Clinic	0.5625	−0.0381 to 1.0309	1.0879	0.1807	Test
PERI2mm	−2.0648	−3.3817 to −1.3268	3.9619	0.1618	Training
PERI2mm	0.1419	−0.4878 to 0.7228	1.67	0.177	Test
Habitat	−1.5201	−2.5308 to −0.8625	3.4889	0.182	Training
Habitat	−0.377	−1.5921 to 0.5052	2.2222	0.1833	Test

## Discussion

The development of a robust preoperative staging model for rectal cancer is crucial to align therapeutic strategies with tumor biology. In this study, a TME-driven MRI model was developed by integrating habitat analysis, peritumoral radiomics, and clinical risk factors. This approach demonstrated superior diagnostic performance compared to standalone models, with the nomogram achieving AUC values of 0.907 and 0.881 in the training and external test cohorts, respectively. The Nomogram performed best in the test set, achieving a balanced accuracy of 0.827, PR-AUC of 0.948, and F1 score of 0.851. Its comprehensive performance significantly outperformed other models, with minimal training-test discrepancy and strong generalization ability. This underscores the clinical value of multidimensional data fusion in resolving staging ambiguities, particularly for T1-2/T3 discrimination—a critical juncture where treatment pathways diverge radically (19). A primary rationale for consolidating T1-2 stages stems from the well-documented limitations of conventional MRI in distinguishing early-stage tumors—manifested by 30% understaging of T2 cancers and 55% overstaging of T1 lesions (20). Overlapping imaging features (e.g., submucosal vs. muscularis propria invasion) contribute to aggregate misclassification rates >25%. In contrast, T3 versus T4 differentiation proves more reliable, utilizing established diagnostic criteria such as invasion of adjacent organs or structures. To address this clinical ambiguity and prioritize actionable stratification, T4 patients were excluded, and T1/T2 stages were consolidated into a single analytical group. This design mirrors strategies proposed in recent consensus guidelines to prioritize actionable staging thresholds (21).

It is noteworthy that tumor length showed opposite associations with T3 stage in univariate (OR = 1.016) and multivariate (OR = 0.963) analyses, though both estimates were close to unity. This reversal likely reflects its modest effect and shared variance with stronger predictors such as mrT stage, CEA, and circumferential growth. Such sign reversal is common when a weak predictor is correlated with other covariates. Therefore, interpretation should prioritize the overall performance of the combined model over individual variables. The nomogram’s strong calibration and AUC (0.907/0.881) confirm its clinical utility.

Habitat analysis emerged as a pivotal component of our model, segmenting tumors into biologically distinct subregions that correlate with invasive potential. This aligns with growing evidence that TME spatial heterogeneity drives tumor aggressiveness (22). For instance, hypoxic habitats have been linked to extracellular matrix remodeling and immune evasion in colorectal cancer (23), while hypervascular regions may indicate angiogenic hotspots associated with extramural spread. While habitat analysis has shown promise in lung and breast malignancies (24, 25), our study pioneers its systematic application to rectal cancer T staging, offering a novel framework to resolve TME-driven spatial complexity (26).

The incorporation of peritumoral radiomics further optimized model performance. The peri-2 mm boundary emerged as the optimal peritumoral region, aligning with evidence that tumor-stroma interactions within this margin reflect early invasive potential. Radiomic features in this zone—such as irregular texture patterns or skewed intensity distributions—likely reflect early desmoplastic reactions or microvascular proliferation preceding macroscopic invasion. Studies have demonstrated that peritumoral radiomics enhances predictive accuracy for preoperative lymph node metastasis in cervical cancer (27) and lymphovascular invasion in rectal cancer (28), aligning with the findings of this research. This suggests the critical clinical value of peritumoral regions, likely attributable to the tumor microenvironment being predominantly enriched at the tumor-stroma interface (29). Furthermore, tumor budding in rectal cancer is localized to the invasive front, characterized by tumor cells detaching from the margin and migrating into the surrounding stroma (30), further supporting the pivotal role of peritumoral areas in T-staging. The fusion of radiomics with clinical parameters outperformed individual models, resonating with Lambin et al. (12), who positioned radiomics as a cornerstone of personalized oncology. The nomogram’s robustness was further reinforced by clinical variables such as elevated CEA, tumor length, and mrT stage, consistent with NCCN guidelines for advanced disease.

Several limitations merit consideration. First, the retrospective design introduces potential selection bias. Second, although multicenter data enhanced generalizability, residual variations in MRI protocols across institutions may persist despite preprocessing standardization. Prospective studies with harmonized imaging parameters are warranted. Finally, the model focused solely on MRI-derived features; integrating CT or PET data could augment insights into metabolic or structural heterogeneity.

In conclusion, this study establishes a TME-driven nomogram that synergizes habitat analysis, peritumoral radiomics, and clinical factors to improve preoperative T staging in rectal cancer. By addressing the limitations of conventional MRI, this model holds significant potential for optimizing therapeutic decision-making, minimizing overtreatment, and enhancing patient outcomes.

## Data Availability

The original contributions presented in the study are included in the article/Supplementary material, further inquiries can be directed to the corresponding authors.
