# Supplementary material for: Application of a MRI model based on tumor microenvironment habitat and peritumoral features in preoperative differentiation of rectal cancer T1-2/T3: a multicenter study
Source: Front Med (Lausanne). 2026 Mar 26;13:1775773. doi: 10.3389/fmed.2026.1775773 (PMC13061711; doi:10.3389/fmed.2026.1775773)
Supplement: Supplementary file 1 [file Data_Sheet_1.docx]

**Supplementary S1.** The main parameters of rectal MRI T2WI

| Hospital | MRI machine | Magnetic field strength | TR | TE | Slice thickness | Interval | FOV | Sequence |
| --- | --- | --- | --- | --- | --- | --- | --- | --- |
| Center A | Siemens Skyra | 3.0T | 5000-7000 | 100-120 | 3-5 | 3.6 | 160×160 | TSE |
|  | GE Signa HDXT | 3.0T | 3500-5000 | 100-120 | 3-5 | 4 | 180×180 | FSE |
| Center B | GE Discovery 750 | 3.0T | 5500-7500 | 105-120 | 3-5 | 3.6 | 200×200 | FSE |
|  | Siemens Skyra | 3.0T | 4000-6000 | 95-120 | 3-5 | 3.6 | 180×180 | TSE |
| Center C | GE Discovery 750 | 3.0T | 5500-7500 | 100-120 | 3-5 | 3.6 | 200×200 | FSE |
|  | GE Signa HDXT | 3.0T | 3500-5000 | 100-120 | 3-5 | 4 | 200×200 | FSE |
| Center D | GE Discovery 750 | 3.0T | 9000-12000 | 60-80 | 4-5 | 1-3 | 280×280 | FSE |

Center A, the Huangdao Hospital of the Affiliated Hospital of Qingdao University; Center B, the Laoshan Hospital of the Affiliated Hospital of Qingdao University; Center C, the Shinan Hospital of the Affiliated Hospital of Qingdao University; Center D, Qingdao Municipal Hospital; TR, repetition time; TE, echo time; FOV, field of view

**Supplementary S2. The excluded features with intra- and inter-ICC＜0.75**

| Feature name | Intra-ICC | Inter-ICC |
| --- | --- | --- |
| wavelet_LHH_firstorder_Mean | 0.1939 | 0.3227 |
| wavelet_HLH_firstorder_Mean | 0.2729 | 0.1371 |
| wavelet_HHL_firstorder_Mean | 0.3647 | 0.4010 |
| wavelet_HHH_glcm_ClusterShade | 0.3846 | 0.3431 |
| wavelet_LHH_firstorder_RootMeanSquared | 0.4462 | 0.4953 |
| wavelet_HHL_firstorder_RootMeanSquared | 0.5214 | 0.5511 |
| wavelet_HHL_glcm_ClusterShade | 0.5306 | 0.5317 |
| wavelet_HLH_firstorder_RootMeanSquared | 0.5784 | 0.4623 |
| original_glszm_SmallAreaLowGrayLevelEmphasis | 0.6621 | 0.6831 |
| original_gldm_SmallDependenceLowGrayLevelEmphasis | 0.6842 | 0.6952 |
| original_glszm_LowGrayLevelZoneEmphasis | 0.7119 | 0.7211 |
| wavelet_HLL_glcm_Correlation | 0.7401 | 0.7643 |
| wavelet_HHH_firstorder_Mean | 0.7434 | 0.7644 |
| log_sigma_2_0_mm_3D_glszm_SmallAreaLowGrayLevelEmphasis | 0.7465 | 0.9887 |
| wavelet_HHH_firstorder_Skewness | 0.7690 | 0.7207 |

**Supplementary S3.** PyRadiomics parameter file

imageType:

Original: {}

LoG:

sigma: [2.0, 3.0, 4.0, 5.0]

Wavelet: {}

featureClass:

shape:

firstorder:

glcm: # Disable SumAverage by specifying all other GLCM features available

- 'Autocorrelation'

- 'JointAverage'

- 'ClusterProminence'

- 'ClusterShade'

- 'ClusterTendency'

- 'Contrast'

- 'Correlation'

- 'DifferenceAverage'

- 'DifferenceEntropy'

- 'DifferenceVariance'

- 'JointEnergy'

- 'JointEntropy'

- 'Imc1'

- 'Imc2'

- 'Idm'

- 'Idmn'

- 'Id'

- 'Idn'

- 'InverseVariance'

- 'MaximumProbability'

- 'SumEntropy'

- 'SumSquares'

glrlm:

glszm:

gldm:

ngtdm:

setting:

normalize: true

normalizeScale: 100 # This allows you to use more or less the same bin width.

interpolator: 'sitkBSpline'

resampledPixelSpacing: [2, 2, 2]

binWidth: 5

voxelArrayShift: 300

label: 1

**Supplementary S4.** Features extracted from each voxel of the tumor VOI

firstorder:

- 'Entropy'

- 'MeanAbsoluteDeviation'

- 'Median'

glcm:

- 'DifferenceAverage'

- 'DifferenceEntropy'

- 'DifferenceVariance'

- 'JointEnergy'

- 'JointEntropy'

- 'InverseVariance'

- 'SumEntropy'

- 'Imc1'

- 'Imc2'

glrlm:

- 'RunEntropy'

- 'RunVariance'

- 'LongRunEmphasis'

glszm:

- 'SizeZoneNonUniformityNormalized'

- 'SmallAreaHighGrayLevelEmphasis'

ngtdm:

- 'Contrast'

- 'Strength'

**Supplementary S5.** CH score of different clusters

| n_clusters | CH score |
| --- | --- |
| 3 | 2235.19 |
| 4 | 1925.14 |
| 5 | 1691.48 |
| 6 | 1524.92 |
| 7 | 1396.02 |
| 8 | 1299.11 |
| 9 | 1231.32 |
| 10 | 1168.95 |

**Supplementary S6**. Hyperparameter Configurations of three classifiers

**1. Logistic Regression (LR)**

| Parameter | Value | Description |
| --- | --- | --- |
| penalty | ‘l2’ | L2 regularization to prevent overfitting. |
| C | 1.0 | Inverse regularization strength. |
| solver | ‘lbfgs’ | Optimization algorithm for loss minimization. |
| max_iter | 100 | Maximum number of iterations for solver convergence. |
| tol | 0.0001 | Tolerance for stopping criterion. |
| fit_intercept | True | Whether to add a bias term. |

**2. Support Vector Machine (SVM) with RBF Kernel**

| **Parameter** | **Value** | **Description** |
| --- | --- | --- |
| kernel | ‘rbf’ | Radial basis function kernel for non-linear classification. |
| C | 1.0 | Regularization parameter. |
| gamma | ‘scale’ | Kernel coefficient, computed as 1/(*n*_features_​⋅*X*.var()). |
| decision_function_shape | ‘ovr’ | One-vs-rest strategy for multi-class classification. |
| tol | 0.001 | Tolerance for stopping criterion. |
| shrinking | True | Use shrinking heuristic to speed up training. |
| max_iter | -1 | No limit on iterations; run until convergence. |

**3. Multi-Layer Perceptron (MLP)**

| **Parameter** | **Value** | **Description** |
| --- | --- | --- |
| hidden_layer_sizes | (100,) | Single hidden layer with 100 neurons. |
| activation | ‘relu’ | Rectified linear unit activation function. |
| solver | ‘adam’ | Adam optimizer for weight updates. |
| learning_rate_init | 0.001 | Initial learning rate. |
| alpha | 0.0001 | L2 regularization strength. |
| max_iter | 200 | Maximum number of epochs. |
| batch_size | ‘auto’ | Mini-batch size, automatically set to min(200, n_samples). |
| tol | 0.0001 | Tolerance for optimization convergence. |
| shuffle | True | Shuffle data at each iteration. |
| early_stopping | False | Early stopping based on validation score not used. |
| beta_1, beta_2, epsilon | 0.9, 0.999, 1e-8 | Adam optimizer hyperparameters (default). |

**Supplementary S7**. Radiomics score is show as follow,

**PERI1mm** label = 0.6338797814207651

-0.030651 * peri_wavelet_LLL_glszm_SmallAreaLowGrayLevelEmphasis

-0.045210 * peri_wavelet_LHH_glcm_MaximumProbability

+0.024278 * peri_wavelet_HHL_firstorder_Skewness

+0.030452 * peri_original_shape_Maximum2DDiameterSlice

+0.004139 * peri_wavelet_LLL_firstorder_10Percentile

-0.027849 * peri_wavelet_HLL_glszm_SmallAreaLowGrayLevelEmphasis

+0.019194 * peri_original_ngtdm_Complexity

-0.033135 * peri_wavelet_LLL_glszm_LargeAreaLowGrayLevelEmphasis

-0.007780 * peri_wavelet_LHH_glszm_SmallAreaLowGrayLevelEmphasis

**PERI2mm** label = 0.6338797814207647 + 0.007645 * peri_wavelet_HLL_glcm_SumEntropy

+0.046651 * peri_wavelet_HHL_firstorder_Skewness

+0.028559 * peri_wavelet_LLL_firstorder_10Percentile

+0.018986 * peri_original_shape_Maximum2DDiameterSlice

+0.057650 * peri_wavelet_LLL_glcm_DifferenceAverage

+0.006044 * peri_wavelet_LHL_glszm_SmallAreaHighGrayLevelEmphasis

-0.023294 * peri_wavelet_LLH_glszm_LargeAreaLowGrayLevelEmphasis

-0.025348 * peri_wavelet_HHL_glszm_ZoneVariance

-0.000518 * peri_original_glcm_MaximumProbability

-0.097186 * peri_wavelet_HLH_ngtdm_Strength

-0.005430 * peri_wavelet_LLL_ngtdm_Strength

-0.032341 * peri_original_glszm_ZoneVariance

+0.000820 * peri_log_sigma_3_0_mm_3D_glcm_ClusterProminence

-0.030736 * peri_wavelet_LHL_glszm_SmallAreaLowGrayLevelEmphasis

+0.033644 * peri_wavelet_LLH_glcm_Idn

+0.026952 * peri_original_shape_MinorAxisLength

+0.021115 * peri_wavelet_HHH_glszm_SmallAreaEmphasis

**PERI3mm** label = 0.6338797814207651

-0.011840 * peri_wavelet_LLL_glszm_SmallAreaLowGrayLevelEmphasis

+0.040264 * peri_wavelet_LLL_firstorder_10Percentile

+0.038019 * peri_wavelet_LLH_glcm_Idn

-0.012068 * peri_wavelet_LLL_glszm_LargeAreaLowGrayLevelEmphasis

-0.081605 * peri_wavelet_HLH_ngtdm_Strength

+0.060463 * peri_wavelet_HHL_firstorder_Skewness

+0.000674 * peri_original_shape_Maximum2DDiameterSlice

-0.009115 * peri_log_sigma_2_0_mm_3D_gldm_LargeDependenceLowGrayLevelEmphasis

-0.000671 * peri_wavelet_LHL_glszm_ZoneVariance

-0.070451 * peri_wavelet_HLL_glcm_MaximumProbability

-0.016660 * peri_wavelet_LLL_firstorder_Skewness

-0.019426 * peri_wavelet_HHL_glcm_Correlation

-0.052648 * peri_original_glszm_ZoneVariance

-0.038473 * peri_log_sigma_3_0_mm_3D_gldm_LargeDependenceLowGrayLevelEmphasis

-0.033781 * peri_wavelet_HLL_firstorder_RootMeanSquared

**Habitat** label =0.6338797814207651 + 0.016222 * wavelet_HLH_glszm_ZoneEntropy_h1

-0.026526 * wavelet_HHL_gldm_LargeDependenceLowGrayLevelEmphasis_h1

+0.013936 * log_sigma_5_0_mm_3D_glcm_Imc1_h2

+0.007578 * log_sigma_3_0_mm_3D_ngtdm_Busyness_h1

-0.006941 * wavelet_LLL_glszm_LargeAreaLowGrayLevelEmphasis_h1

-0.012486 * wavelet_LHL_gldm_LargeDependenceLowGrayLevelEmphasis_h2

-0.035073 * log_sigma_3_0_mm_3D_glcm_MaximumProbability_h3

-0.047337 * original_shape_Sphericity_h2

-0.047669 * wavelet_HLL_glcm_MaximumProbability_h2

+0.027368 * log_sigma_4_0_mm_3D_firstorder_Kurtosis_h2

**Supplementary S8. Delong test of the four models**

**
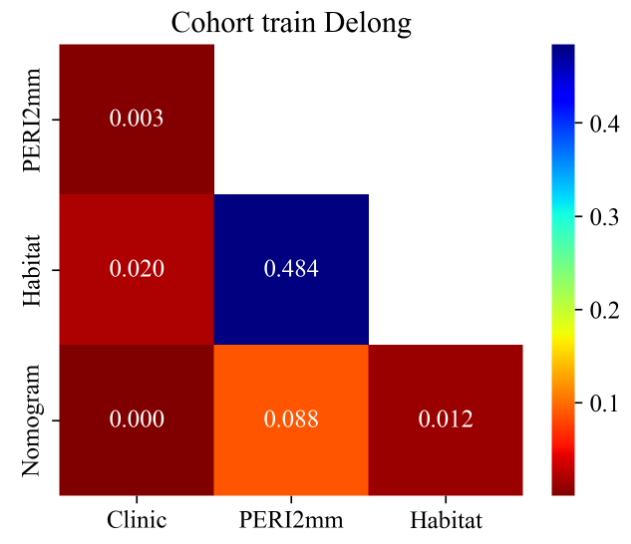

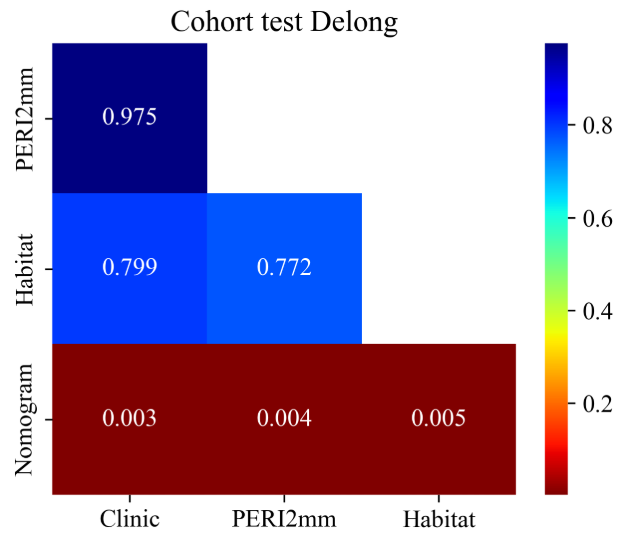
**

**Supplementary S9.** Univariate analysis of clinical characteristics

| feature | Log(OR) | lower 95%CI | upper 95%CI | OR | OR lower 95%CI | OR upper 95%CI | P value |
| --- | --- | --- | --- | --- | --- | --- | --- |
| sex | 0.602 | 2.780e-01 | 9.250e-01 | 1.825 | 1.320 | 2.522 | 0.002 |
| age | 0.009 | 5.000e-03 | 1.300e-02 | 1.009 | 1.005 | 1.013 | 0.000 |
| DIS | 0.007 | 4.000e-03 | 1.000e-02 | 1.007 | 1.004 | 1.01 | 0.000 |
| length | 0.016 | 1.000e-02 | 2.100e-02 | 1.016 | 1.010 | 1.021 | 0.000 |
| Cir | 1.450 | 1.031 | 1.869 | 4.263 | 2.804 | 6.482 | 0.000 |
| mrT | 0.263 | 1.710e-01 | 3.550e-01 | 1.301 | 1.186 | 1.426 | 0.000 |
| depth | 0.287 | 1.860e-01 | 3.880e-01 | 1.333 | 1.204 | 1.474 | 0.000 |
| mrN | 0.998 | 6.470e-01 | 1.349 | 2.713 | 1.910 | 3.854 | 0.000 |
| M | 29.438 | -1.530e+06 | 1.530e+06 | 6.095e+12 | 0.000 | inf | 1.000 |
| MRF | 3.136 | 1.455 | 4.816 | 2.300e+01 | 4.284 | 123.47 | 0.002 |
| EMVI | 2.565 | 1.358 | 3.772 | 1.300e+01 | 3.888 | 43.467 | 0.000 |
| CEA | 1.645 | 1.103 | 2.187 | 5.182 | 3.013 | 8.908 | 0.000 |
| CA199 | 33.666 | -9.610e+06 | 9.610e+06 | 4.177e+14 | 0.000 | inf | 1.000 |

**Supplementary S10.** Multivariate analysis of clinical characteristics

| feature | Log(OR) | lower 95%CI | upper 95%CI | OR | OR lower 95%CI | OR upper 95%CI | P value |
| --- | --- | --- | --- | --- | --- | --- | --- |
| DIS | -0.003 | -0.013 | 0.007 | 0.997 | 0.987 | 1.007 | 0.609 |
| age | -0.031 | -0.053 | -0.009 | 0.970 | 0.948 | 0.991 | 0.020 |
| length | -0.038 | -0.068 | -0.007 | 0.963 | 0.934 | 0.993 | 0.044 |
| mrT | 1.109 | 0.559 | 1.659 | 3.031 | 1.749 | 5.254 | 0.001 |
| depth | 0.122 | -0.038 | 0.282 | 1.130 | 0.963 | 1.326 | 0.211 |
| sex | -0.158 | -0.794 | 0.477 | 0.854 | 0.452 | 1.611 | 0.682 |
| mrN | 0.157 | -0.459 | 0.774 | 1.170 | 0.632 | 2.168 | 0.675 |
| Cir | 1.134 | 0.364 | 1.904 | 3.109 | 1.439 | 6.713 | 0.015 |
| CEA | 1.452 | 0.639 | 2.265 | 4.270 | 1.895 | 9.631 | 0.003 |
| EMVI | 0.884 | -0.622 | 2.391 | 2.422 | 0.537 | 10.924 | 0.334 |
| MRF | 0.769 | -1.183 | 2.721 | 2.158 | 0.306 | 15.196 | 0.517 |
